# Can novel Apo A-I polymorphisms be responsible for low HDL in South Asian immigrants?

**DOI:** 10.4103/0971-6866.42321

**Published:** 2008

**Authors:** Sunita Dodani, Yanbin Dong, Haidong Zhu, Varghese George

**Affiliations:** Medical College of Georgia, Augusta, GA, USA

**Keywords:** Coronary artery diseases, high density lipoprotein, lipids, risk factors, South Asians

## Abstract

Coronary artery disease (CAD) is the leading cause of death in the world. Even though its rates have decreased worldwide over the past 30 years, event rates are still high in South Asians. South Asians are known to have low high-density lipoprotein (HDL) levels. The objective of this study was to identify Apolipoprotein A-I (Apo A-I) polymorphisms, the main protein component of HDL and explore its association with low HDL levels in South Asians. A pilot study on 30 South Asians was conducted and 12-h fasting samples for C-reactive protein, total cholesterol, HDL, low-density lipoprotein (LDL), triglycerides, Lipoprotein (a), Insulin, glucose levels, DNA extraction, and sequencing of Apo A-I gene were done. DNA sequencing revealed six novel Apo A-I single nucleotide polymorphisms (SNPs) in South Asians, one of which (rs 35293760, C938T) was significantly associated with low (<40 mg/dl) HDL levels (*P* = 0.004). The association was also seen with total cholesterol (*P* = 0.026) and LDL levels (*P* = 0.032). This pilot work has highlighted some of the gene-environment associations that could be responsible for low HDL and may be excess CAD in South Asians. Further larger studies are required to explore and uncover these associations that could be responsible for excess CAD risk in South Asians.

## Introduction

Coronary artery disease (CAD) is the leading cause of morbidity and mortality in the world. Even though rates have decreased in the United States (US) and other developed countries over the past 30 years, event rates are still high in South Asians,[1] people with ancestors from the Indian subcontinent (i.e., India, Pakistan, Bangladesh, Nepal, Bhutan, and Sri Lanka). CAD risk factors are observed in South Asians at a younger age, predisposing them to a higher risk for CAD than other populations.[2–4] Though, South Asians are the second fastest growing Asian immigrant group in the US, the causes of the increased risk of CAD remains unclear.[1]

The available literature clearly shows that South Asians have a much higher prevalence of diabetes, insulin resistance parameters, dyslipidemias [lower high-density lipoprotein (HDL), increased lipoprotein [a] (Lpa), and higher triglycerides (TGs)], and low levels of physical activity.[5–12] However, even taking these differences into account, traditional risk factors may not fully explain the increased risk for CAD in South Asians, especially in South Asians immigrants.[4,12–15] Migration is clearly an important factor in determining the increased risk of CAD; however, other migrating populations (e.g., Afro-Caribbean) have not shown an increased risk of CAD compared with the indigenous population.[8]

Among the numerous genetic and lifestyle factors associated with CAD, dyslipidemias are one of the most important. HDL cholesterol plays a protective role and low HDL is an independent risk factor for CAD.[16] This protective effect of HDL is related to its role as an antiatherogenic, antioxidant, and anti-inflammatory agent that prevents low-density lipoprotein (LDL) oxidation, primarily through Apolipoprotein A-I (Apo A-I), the main protein of HDL. Apo A-I is found to be strongly correlated with HDL level and function.[17,18]

Several studies have shown the evidence that low HDL level is associated with increased risk of CAD in South Asians.[19,20] In addition, South Asians also have a higher concentration of small, less protective HDL particles, also observed in the Framingham offspring study, contributing to the higher CAD risk in South Asians.[20,21] These smaller HDL particles contain two Apo A-I molecules compare to four molecules in larger HDL particle. Although there are several causes of low HDL levels, e.g., increased catabolism, decreased synthesis, and altered equilibrium of HDL between intravascular and extravascular spaces, the major cause has been found to be decreased or absent synthesis of Apo A-I.[22] It has also been shown that plasma levels of Apo A-I and Apo-B provide better markers for predicting the presence of CAD risk as compared to traditional lipid measures and the levels of these apolipoproteins are lower in the South Asians as compared to Western populations.[23]

### Apolipoprotein A-I: Structure and function

Apo A-I (APOAI gene, Apo A-I protein) is the major protein of HDL and consists of 243 amino acid long peptide, synthesized mainly in the liver and to some extent in the small intestine. The inverse relationship between HDL plasma levels and CAD has been attributed to the role that HDL and its major constituent Apo A-I play in reverse cholesterol transport (RCT). The efficiency of RCT depends on the specific ability of Apo A-I to promote cellular cholesterol efflux, bind lipids, activate lecithin: cholesterol acyltransferase (LCAT), and form mature HDL that interact with specific receptors and lipid transfer proteins.[24,25] The APOAI gene is present along with the APOC3 and APOA4 genes, on chromosome 11(11q23.3-qter).[26] It has also been shown that the A allele of the APOAI gene contributes to the severity of CAD and low levels of HDL among Northern Indians.[27]

South Asians are commonly known to have low HDL levels; however, its association with Apo A-I gene polymorphisms has not been fully examined. We predict that South Asians have specific Apo A-I polymorphisms that may be related to low HDL levels and increased CAD risk. The objective of the current study was to identify Apo A-I polymorphisms and their association with low HDL levels and other risk factors for CAD in South Asians living in the United States.

## Materials and Methods

A pilot cross-sectional study on 30 subjects between the ages of 40 and 65 years with equal number of males and females was conducted. After institutional review board approval, subjects were recruited from two of the main Hindu temples in Georgia and informed written consent was obtained. Information on socio-demographic and lifestyle characteristics traditional, and specific CAD risk factors [Tables 1 and 2] was obtained. A 12-h fasting blood samples were for C-reactive protein (CRP), total cholesterol, HDL, LDL, TG, Lpa, Insulin, Apo A-I, and glucose levels. Blood samples were centrifuged to obtain buffy coat for DNA extraction and sequencing of Apo A-I gene.

**Table 1:** Demographic characteristics of study group (*N* = 29)

Variable	*n* (%)
*Age*	56^a^ (6.47^b^)
Gender	
Male	14 (48.3)
Female	15 (51.7)
*Ethnicity*	
Gujarati	6 (20.7)
Hindi	15 (51.7)
South Indian	5 (17.2)
Bengali	1 (3.4)
Punjabi	1 (3.4)
Marathi	1 (3.4)
*Work Type*	
Medical Doctor	2 (6.9)
Business	3 (10.3)
Government Job	8 (27.6)
Engineer	5 (17.2)
Housewife	4 (13.8)
Others	
*Education*	
Undergraduate	7 (24.1)
Graduate	9 (31.0)
Postgraduate	13 (44.8)

a Mean

b Standard deviation

**Table 2:** Coronary artery disease risk factors in study group

Variable	*n*/*N* (%)	Mean ± Std.
Cholesterol		207.36 ± 41.65
Desirable (<200)	13/28 (44.8)	174.23 ± 16.35
Borderline high (200-239)	10/28 (34.5)	215.10 ± 11.78
High (≥240)	5/28 (17.2)	278.000 ± 25.31
HDL		51.29 ± 8.74
Low (<40)	4/28 (13.8)	37.75 ± 1.89
Normal (40-59)	18/28 (62.1)	50.50 ± 5.31
High (≥60)	6/28 (20.6)	62.67 ± 3.27
LDL		134.43 ± 40.55
Optimal (<100)	6/28 (20.7)	90.50 ± 7.34
Above optimal (100-129)	8/28 (27.6)	115.88 ± 8.71
Borderline high (130-159)	8/28 (27.6)	139.25 ± 6.67
High (160-189)	3/28 (10.3)	169.00 ± 3.00
Very high (≥190)	3/28 (10.3)	224.33 ± 21.50
Triglycerides		108.11 ± 46.05
Normal (<150)	23/28 (79.3)	90.52 ± 25.63
Borderline high (150-199)	3/28 (10.3)	170.33 ± 10.41
High (200-249)	2/28 (6.9)	217.00 ± 11.31
Lp(a) lipoprotein		31.07 ± 28.90
Normal (<39)	21/28 (72.4)	19.62 ± 10.79
Borderline high (39-49)	3/28 (10.3)	45.67 ± 5.77
High (50-59)	3/28 (10.3)	56.00 ± 4.36
Very high (≥60)	1/28 (3.4)	153.00
BMI categories		24.93 ± 2.46
Normal (18.5-23.0)	6/29 (20.7)	21.73 ± 1.41
Pre-obese (23.1-29.9)	23/29 (79.3)	25.77 ± 1.93
Waist circumference		90.97 ± 15.60
Males	14/29 (48.3)	95.14 ± 19.85
>90	6/14 (42.9)	106.00 ± 27.66
≤90	8/14 (57.1)	87.00 ± 2.93
Females	15/29 (51.7)	87.07 ± 9.32
>85	8/15 (53.3)	94.13 ± 5.99
≤85	7/15 (46.7)	79.00 ± 4.28
Hypertension		
Yes	5/29 (17.2)	
No	24/29 (82.8)	
Diabetes		
Yes	2/29 (6.9)	
No	27/29 (93.1)	
Family history of CAD^a^		
Yes	14/29 (48.3)	
No	15/29 (51.7)	
Physical activity^a^		
Yes	26/29 (89.7)	
No	3/29 (10.34)	

BMI, body mass index; HDL, high-density lipoprotein; LDL, low-density lipoprotein; CAD, coronary artery disease

a Family history of CAD and physical activity was defined according to standard criteria[49,50]

### DNA extraction from blood specimens

Each blood buffy coat sample (750 µl) was assigned a unique DNA identification number. Genomic DNA was extracted from 250 µl buffy coat using column purification system (Qiagen^®^ QiAamp^®^ Blood Mini Kit,QIAGEN, Valencia, CA, USA), which yields 4-12 µg of DNA according to the manufacturer. The remaining specimen was frozen at −80°C. An aliquot of DNA was diluted and the absorbance at λ 260 nm and λ 280 nm measured using an Eppendrof Biophotometer for verification of quality and concentration. DNA were diluted to 50 ng/µl and stored at −80°C.

### DNA sequencing of Apo A-I gene

One microgram of genomic DNA samples in 20 µl H_2_O was sent to Seq Wright DNA Technology Service (Houston, TX, USA) for sequencing. Seq Wright is compliant with 21 CFR 58 (GLP) and 21 CFR 11 (Electronic Signatures) and has implemented elements of 21 CFR 210/211 (cGMP). Upon receiving genomic DNA samples, an initial qualitative and quantitative assessment was performed by inspection on agarose gel or optical density. To sequence the Apo A-I gene (11q23, NM_000011.8, 1869 bp), four pairs of sequencing primers were designed to allow sufficient overlap in individual sequencing reads. Primers used for polymerase chain reaction (PCR) were also used for sequencing. Fluorescent dye-terminator chemistry was used for bi-directional DNA sequencing, using ABI Prism™ 3730 xl DNA sequencer, which typically gives >650 bp Q20/Phred20 read lengths. Mutations/heterozygote/heter-indels were scored by automated comparative analysis against the NM_000011.8 sequence. The results were reviewed to ensure their accuracy.

### Data analysis

Data were entered and analyzed using window-based SAS system (version 9.1). A detailed descriptive statistics were conducted to explore distribution of CAD risk factors, including demographics and other relevant variables. The genotype for each person at the polymorphic loci of Apo A-I gene was determined. Association tests of genetic polymorphisms of the Apo A-I gene was performed using both genotypes and alleles. For most single nucleotide polymorphisms (SNPs) with two alleles, a 2 × 3 contingency table was constructed for association tests with genotypes and a 2 × 2 contingency table for association tests with alleles [Table 3]. Fisher-exact test was performed to assess association of Apo A-I polymorphism with CAD risk factors.

**Table 3:** Apo A-I SNPs in study subjects (*N* = 20)

Apo A-I SNPs	Major allele frequency		*P*-value*
		
	HDL <40 mg	HDL ≥40 mg	
Rs5070 (T319C)	80.0	63.3	0.529
rs17249470 (T655C)	80.0	66.7	0.67
rs17249463 (T756C)	80.0	66.7	0.67
rs35293760 (C938T)	40.0	96.7	0.004
rs7116797 (T1001C)	70.0	66.7	0.595
rs5075 (C1149T)	90.0	100.0	0.075

SNPs, single nucleotide polymorphisms; Apo A-I, apolipoprotein A-I; G, genotype

* Fisher-exact test.

## Results

Of the 30 subjects, one subject could not complete the study questionnaire and blood work, and was excluded from the study. We were unable to draw blood sample from a subject, but other information was obtained so the subject was included in the study. Due to financial constraints and limited available funding, DNA sequencing was performed on 20 subjects.

The mean age of subjects was 56 ± 6.46 years with an almost equal number of males and females. The prevalence of CAD risk factors was (a) hypertension 17%; (b) diabetes 6.9%; (c) high cholesterol (≥200 mg/dl) 34.5%; (d) HDL <40 mg 13.8%; and (e) positive family history of cardiovascular disease 48.3% [Table 2]. A total of 41.45% subjects were overweight and no one was a current smoker.

**Figure 1:**
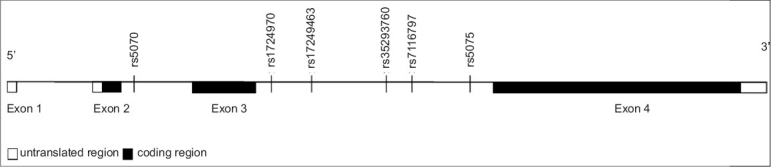
The structure of the ApoA-I gene. Exons are shown as boxes, introns, and intergenic regions as lines. The six SNPs identified are located at intron 2 and 3

The DNA analysis for Apo A-I polymorphisms revealed six novel SNPs [Figure 1] in South Asian samples. One of the SNPs rs35293760 (C938T) was found to be significantly associated with low (<40 mg/dl) HDL levels [*P* = 0.004; Table 3]. The association was also seen with total cholesterol (*P* = 0.026) and LDL levels [*P* = 0.032; Table 4].

**Table 4:** Association of (C938T) with coronary artery disease risk factors (*N* = 20)

Variable		C938		
	
	*C*	*T*/*C*	*P* value*
Age	n	n	
<50 years	3	1	
≥50 years	13	3	1.000
Cholesterol			
<200	5	4	
≥200	11	0	0.026
HDL index			
<0.99	10	2	
≥1.00	6	2	1.000
LDL			
<100	2	3	
≥100	14	1	0.032
Triglycerides			
<150	14	2	
≥150	2	2	0.162
Lp(a) lipoprotein			
<39	11	2	
≥39	5	2	0.587

## Discussion

Apo A-I is the major protein component of HDL and known to be associated with HDL levels and its function. Epidemiologic studies have shown that HDL and Apo A-I levels are inversely correlated with the risk of developing CAD.[18,28,29] Although various factors such as genetic variations, diet, exercise, alcohol, smoking, hormones, and certain drugs can significantly influence the levels of HDL and Apo A-I,[30] family and twin studies have demonstrated a strong genetic heritability, accounting for up to 66% of the variability of HDL, and Apo A-I levels.[31,32] Furthermore, 40-60% of the interindividual variation in HDL concentration is controlled at the genetic level[31] and the strong positive correlation between plasma levels of Apo A-I and HDL suggests that Apo A-I gene polymorphisms may be linked to variability in HDL levels as well as to dysfunction.[28,31]

To the best of our knowledge, this is the first study discovering novel polymorphisms of Apo A-I and assessing their association with low HDL in South Asians. Chabra and colleagues found the Apo A-I G-75A mutation associated with CAD and lower levels of HDL in Northern Indians; this is the sole study in a small group of Northern Indians.[27] However to our knowledge, we have found six novel SNPs in South Asian immigrants that have not been seen in any of the previous studies.

The findings of diabetes, hypertension, and low HDL prevalence were consistent with previously reported data.[13,33–36] Studies have shown that polymorphism in the Apolipoprotein C-III gene promoter was associated with metabolic syndrome in South Indians.[37] A recent small study conducted on Pakistanis suggested that the promoter region of the Apo A-I gene may play a role in determining blood pressure.[38]

Different polymorphisms in genes coding for proteins related to lipid metabolism may influence the HDL concentration.[39–41] The G/A polymorphism of the Apo A-I promoter region is one of the most widely investigated.[42–48] In our study, one of the SNP, i.e., rs35293760 (C938T) showed strong association with low HDL (*P* = 0.004) that may further increases CAD risk in South Asians; however, further studies are required to explore these associations in different South Asian groups. Moreover, the association was also found with total cholesterol and LDL [Table 4], suggesting dyslipidemias as a whole and not just low HDL levels predisposing South Asians to increased risk of CAD. The association of rs35293760 (C938T) with cholesterol was seen in subjects who had low cholesterol levels. Associations with other CAD risk factors were not found and could be due to small sample size.

All six SNPs identified from the current study are intronic SNPs. The rs35293760 (C938T) is located at intron 3 region [Figure 1]. The function of the rs35293760 (C938T) is not clear. Introns are located between protein-coding exons. The intronic region was initially thought to be a huge genetic waste in gene transcripts. Recently, this misconception was corrected by the observation of intronic microRNA. miRNA is usually 18-25 oligonucleotides in length and is capable of either directly degrading its intracellular messenger RNA (mRNA) target or suppressing the protein translation of its targeted mRNA.[49,50] The rs35293760 (C938T) could be in the miRNA region, or could be in linkage disequilibrium with another causal mutation.

This study has provided some of the novel preliminary results; however, several limitations of this study must be considered. First, this was a cross-sectional pilot study and, as in all such studies, the data are exploratory and do not allow the establishment of causality and may not account for changes over time. Second, we recruited participants from local Hindu temples and therefore participants may not be completely representative of the South Asian community. However, people attending these temples were from mixed ethnic backgrounds, and data were collected from participants who attended weekend worship services, which in general are attended by South Asians from different and diverse ethnic groups. Therefore, we anticipate the selection bias is minimal and that the sample is representative of South Asians immigrants living in Georgia. Last but not least, due to the limited budget, this was a small sample size and we were not able to make any definitive conclusions regarding the association of Apo A-I with many of the CAD risk factors. A proposal for a larger case-control study has already been submitted to the National Institute of Health.

## Conclusions

Despite the limitations noted above, the present study demonstrates a number of important findings. We highlighted the importance of Apo A-I and its role in maintaining HDL level and function. Genetic or other novel risk factors may modulate HDL levels and thus may render South Asians susceptible to CAD. Discovery of novel polymorphisms like this will help to understand further the causes of excess CAD risk in South Asians so that preventative strategies targeted especially to this high-risk group can be developed. To obtain definitive relationship and association of these novel polymorphisms with CAD and its risk factors, larger longitudinal studies are required with large South Asian sample. The current pilot work has provided sufficient information for a larger study to understand further the association of Apo A-I polymorphisms with CAD and its risk factors in multiethnic South Asian.
